# A narrative review of facilitators and barriers to smoking cessation and tobacco-dependence treatment in patients with tuberculosis in low- and middle-income countries

**DOI:** 10.18332/tid/125195

**Published:** 2020-08-06

**Authors:** Kamila Zvolska, Alexandra Pankova, Iveta Nohavova, Rumana Huque, Helen Elsey, Melanie Boeckmann, Aziz Sheikh, Kamran Siddiqi, Eva Kralikova

**Affiliations:** 1 Centre for Tobacco-Dependent, Third Department of Medicine, Department of Endocrinology and Metabolism, First Faculty of Medicine, Charles University, Prague, Czech Republic; 2 Institute of Hygiene and Epidemiology, First Faculty of Medicine, Charles University, Prague, Czech Republic; 3 Department of Research and Development, ARK Foundation, Dhaka, Bangladesh; 4 Department of Health Sciences, University of York, York, United Kingdom; 5 Department of Environment and Health, School of Public Health, Bielefeld University, Bielefeld, Germany; 6 Institute of General Practice, Addiction Research and Clinical Epidemiology Unit, Medical Faculty, Heinrich-Heine-University, Düsseldorf, Germany; 7 Usher Institute, University of Edinburgh, Edinburgh, United Kingdom

**Keywords:** tuberculosis, developing countries, tobacco use disorder, smoking cessation, barriers and facilitators

## Abstract

**INTRODUCTION:**

Smoking is a substantial cause of premature death in patients with tuberculosis (TB), particularly in low- and middle-income countries (LMICs) with high TB prevalence. The importance of incorporating smoking cessation and tobacco-dependence treatment (TDT) into TB care is highlighted in the most recent TB care guidelines. Our objective is to identify the likely key facilitators of and barriers to smoking cessation for patients with TB in LMICs.

**METHODS:**

A systematic search of studies with English-language abstracts published between January 2000 and May 2019 was undertaken in the EMBASE, MEDLINE, EBSCO, ProQuest, Cochrane and Web of Science databases. Data extraction was followed by study-quality assessment and a descriptive and narrative synthesis of findings.

**RESULTS:**

Out of 267 potentially eligible articles, 36 satisfied the inclusion criteria. Methodological quality of non-randomized studies was variable; low risk of bias was assessed in most randomized controlled studies. Identified facilitators included brief, repeated interventions, personalized behavioural counselling, offer of pharmacotherapy, smoke-free homes and a reasonable awareness of smoking-associated risks. Barriers included craving for a cigarette, low level of education, unemployment, easy access to tobacco in the hospital setting, lack of knowledge about quit strategies, and limited space and privacy at the clinics. Findings show that the risk of smoking relapse could be reduced through consistent follow-up upon completion of TB therapy and receiving a disease-specific smoking cessation message.

**CONCLUSIONS:**

Raising awareness of smoking-related health risks in patients with TB and implementing guideline-recommended standardized TDT within national TB programmes could increase smoking cessation rates in this high-risk population.

**ABBREVIATIONS BSS:** behavioural support session, DOTS: directly observed treatment short-course (also known as TB-DOTS), HIC: high-income countries, HW: health workers, LHW: lay health workers, LMICs: low- and middle-income countries, MA: medical assistants, MI: motivational interviewing, NGO: non-governmental organisations, NRT: nicotine replacement therapy, RCS: randomized controlled studies, SCI: smoking cessation intervention, SCIDOTS: directly observed therapy shortcourse plus smoking cessation intervention, SLT: smokeless tobacco, TB: tuberculosis, TDT: tobacco dependence treatment, WHO: World Health Organization.

## INTRODUCTION

The ‘dual epidemics’ of pulmonary tuberculosis (TB) and tobacco smoking are major global public health challenges faced especially by low- and middle-income countries (LMICs)^1,2^. Tobacco smoking increases the risk of pulmonary TB infection, development of pulmonary TB and related mortality, and is closely associated with multi-drug resistant TB^3^. Smoking is positively associated with poor TB treatment outcomes, especially treatment failures and early deaths^4^.

Abstinence from smoking is essential for patients with TB. TB mortality rates drop substantially after quitting smoking^5^; interestingly, however, Jeyashree et al.^6^ did not find any randomized controlled trials to support the effect of smoking cessation on TB treatment outcomes. Two comprehensive practice and policy guidelines on tobacco cessation interventions within TB programmes were developed by the World Health Organization (WHO) and the International Union Against Tuberculosis and Lung Disease (IUATLD)^1,3^. These guidelines highlight the importance of incorporating professional tobacco-dependence treatment (TDT) into TB care. While only about 4% of unassisted quit attempts in the general population are successful^7^, simple advice from a clinician to patients with TB who smoke has been shown to increase abstinence rates significantly (by 30%) compared to no advice^1^. Although in general populations, behavioural support combined with pharmacotherapy is the most effective strategy in helping people to quit, there is no evidence for the effectiveness of this strategy in TB patients who smoke. While medications are widely recommended, their costs prohibit their use in LMICs^8^.

A systematic review of the effectiveness of smoking cessation interventions among TB patients was already published, but little is known about the factors that affect smoking cessation and TDT in these patients. Thus, we conducted a narrative literature review with the main aim to identify facilitators and barriers that affect smoking cessation and TDT among people with TB in LMICs.

## METHODS

### Search strategy

**Figure 1:**
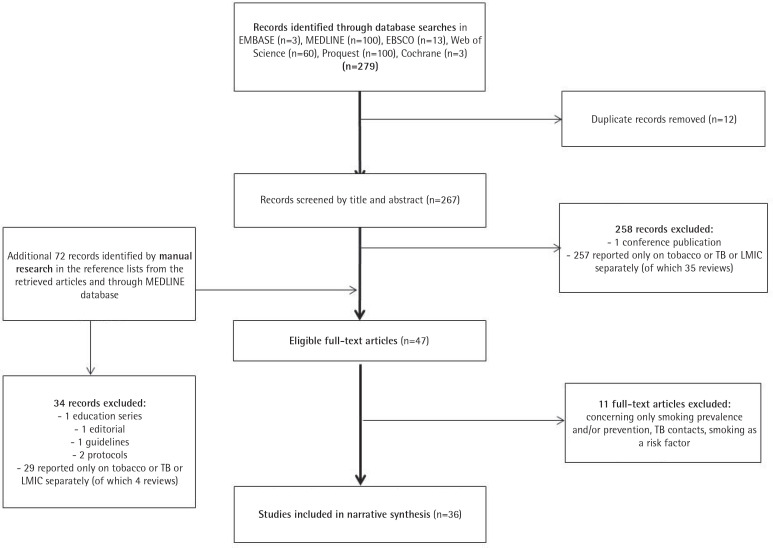
PRISMA flow diagram illustrating the literature selection process

A systematic search was conducted by the Institute of Scientific Information of the First Faculty of Medicine, Charles University in Prague, Czech Republic. The search was carried out in May 2019 and was followed by manual literature searches in the reference lists from the retrieved articles and through MEDLINE database to identify referenced articles and additional articles on smoking cessation in the TB context in LMICs^9^. Records were identified through searches in: 1) Ovid–EMBASE and MEDLINE databases selected, 2) EBSCO databases – Academic Search Complete database selected, 3) ProQuest, 4) Cochrane, and 5) Web of Science databases; and included articles published between January 2000 and May 2019 according to the inclusion and exclusion criteria described below (see the search strategy in the Figure 1 flow diagram). Search terms and combinations thereof included: [facilitators OR barriers OR effectiveness OR success rate OR factors OR outcome(s) OR abstinence] AND [low-income countries OR middle-income countries OR specific countries according to the World Bank] AND [tobacco OR tobacco use cessation OR smoking OR smoking cessation OR treatment] AND [TB OR tuberculosis OR TB programme].

### Research question

What are the facilitators and barriers for people with TB to quit smoking in LMICs?

### Inclusion and exclusion criteria

Inclusion criteria of present review were: English-language abstract of the article (irrespective of the language used in the main text), study population focused at people with TB in LMICs (according to the World Bank)^9^ who smoke (self-reported or biochemically validated smoking status) and were aged ≥15 years. We excluded studies on only smoking prevention and prevalence, those of not original research, and if no full text was available.

### Study selection

The eligibility of studies was assessed using the inclusion criteria by two investigators (EK, KZ). A third investigator (AP) resolved any discrepancies in the opinions of the two investigators, checked by IN, and comments included from RH, HE, AS and KS.

### Data extraction and quality assessment

Data extraction was conducted by one reviewer to be subsequently discussed and checked by at least one other reviewer. Extracted data included the following items: author/year, country, study design, sample size, study purpose, findings, limitations, and quality assessment. Quality assessment was performed after data extraction and done independently by two reviewers (KZ, EK). Randomized controlled studies (RCS) were assessed using the Cochrane Risk of Bias tool for intervention studies^10^, non-randomized studies using the Newcastle-Ottawa Scale (NOS) and modified NOS adapted for cross-sectional studies^11^.

### Data synthesis and reporting

We descriptively summarised data and undertook a narrative synthesis of findings. PRISMA was used to inform reporting.

## RESULTS

The initial search generated 267 potentially eligible articles after duplication removal, plus 72 records identified additionally by manual search (n=339) (Figure 1, PRISMA flow diagram)^12^. Only 36 publications were deemed relevant for review after screening abstracts and full texts according to the pre-specified inclusion and exclusion criteria. Of those papers excluded, 94.4% (286/303) reported only on tobacco or TB or LMICs separately, 11 (3.6%) were excluded for being prevention or prevalence studies, or for assessing smoking as a risk factor, with the remainder (2%) not being original research.

### Characteristics of eligible studies

Regarding design, the selected studies were prospective (including randomized controlled and non-randomized studies), cross-sectional studies, and qualitative studies. All studies but one (in French) were published in English. Eight of the included studies were RCS^13-20^, 28 articles reported on non-randomized studies^21-48^. Characteristics of the studies and their main findings are presented in Tables 1 and 2. Only one study recruited patients aged ≥15 years, with the participants of the other studies aged ≥18 years. Studies were conducted mainly in TB clinics, centres or units, health centres registered as diagnostic by TB program or provided DOTS services, respiratory clinics and, less often, in primary healthcare services. One study intervention was provided at home. In addition to smoking of cigarettes followed in all studies, 1 study included also rolled tendu or temburni leaf (bidi) smoking, 2 studies a hookah session, and 7 studies the use of smokeless tobacco. Data extracted from the studies were reviewed to identify possible facilitators of and barriers to smoking cessation or TDT and relapse (Table 3). Table 4 shows factors associated with smoking relapse among TB patients.

**Table 1:** Summary of included randomized controlled studies concerning smoking cessation/TDT in patients with TB of LMICs (N=8)

*Author, year*	*Location*	*Study design*	*Sample size/ age/sex/type of sample*	*Study purpose*	*Findings*	*Limitations*
Aryanpur et al.^13^ (2016)	Iran	Randomized controlled study	n=183 newly-diagnosed (confirmed) patients who currently smoke with pulmonary TB, aged >18 years. Setting: health centres implementing the Directly Observed Treatment Short Course (DOTS) strategy.	Success rate at 6 months.	Combined intervention including individualized counselling sessions of quitting (behavioural therapy) plus medical treatment with slow- release bupropion can lead to a significant increase (71.7%) in the rate of continuous smoking cessation at the end of sixth months.	The outcome of smoking cessation was measured until the end of TB treatment.
Elsey et al.^14^ (2015)	Pakistan	Cluster randomized controlled study, a secondary analysis (data from the ASSIST trial)	n=1573 patients aged >18 years who were regular smokers of more than 1 cigarette or hookah session a day. Setting: health centres registered as diagnostic centres by the TB program.	To identify predictors of continuous and short-term abstinence, and continued smoking in patients suspected of TB.	A confirmed TB diagnosis was a significant predictor of continuous abstinence at 25 weeks, gender does not predict cessation, socio-familial context particularly at work may undermine smokers’ ability to achieve continuous abstinence, having a child at home was not a significant predictor of abstinence.	see Siddiqi et al.^20^ (2013)
Goel et al.^15^ (2017)	India	Cluster randomized controlled study	n=152 sputum smear-positive pulmonary TB patients, aged ≥15 years, who were current and occasional smokers; 96.7% men, aged 30–44 years. Setting: TB units	To assess treatment success (= the sum of cured, smear- or culture-negative patients in the last month of treatment) and to assess smoking cessation rates (patient had not smoked at all in the last 2 weeks).	Difference in TB treatment outcome was found to be insignificant between intervention (ABC intervention from TB health visitor) and control arm. Treatment success was higher among quitters (74.7%) compared to smokers (25.2%), the difference was found to be significant (IRR=1.31; 95% CI: 1.30–1.32; p≤ 0.0001). At the end of treatment, 57 (80.2%) patients in the intervention arm had quit smoking against 42 (57.5%) in the control arm. After adjusting for confounders, the relative risk of quitting during followup was significantly higher in the intervention arm compared to the control arm for both PP analysis (adjusted IRR=1.56; 95% CI: 1.24–1.93; p<0.0001).	Insignificant difference in TB treatment outcome could be due to the fact that for an intervention to bring positive treatment outcome, the total time spent for each session should be relatively more than it was in the study.
Kumar et al.^16^ (2017)	India	Randomized clinical study	n=73 patients aged >18 years, with TB and with a history of current smoking (80 with HIV infection and 80 with TB). Setting: clinic	To determine the efficacy of physicians’ advice in addition to standard counselling compared with standard counselling alone in quitting smoking.	Among the patients with TB receiving physician’s advice using a modified version of the 5As strategy for smoking cessation plus a brochure containing smoking cessation information with standard counselling, 44.4% (16/36) quit smoking, while 40.5% (15/37) quit smoking with counselling from the counsellors alone plus a brochure containing smoking cessation information (p=0.735).	Only a 1-month abstinence period was assessed.
Louwagie et al.^17^ (2014)	South Africa	Randomized controlled intervention study	n=409 newly diagnosed adult patients with TB identified as current smokers. Setting: primary care public facilities	To determine the efficacy of brief motivational interviewing by lay health workers (LHW) in assisting TB patients with tobacco cessation in a setting with high HIV-TB coinfection rates.	Motivational interviewing (MI) by lay counsellors to promote smoking cessation in TB patients approximately doubled sustained biochemically verified smoking abstinence for at least 6 months compared with brief advice alone [24/83 (28.9%) vs 11/83 (13.3%)].	MI was offered in only a single brief session by LHCWs. LHCWs delivered MI without monitoring by video-taping. Followup measurement was not blinded. Pharmacotherapy was not offered to smokers because of local unavailability. Potential over-reporting of self-reported quit rates.
Louwagie & Ayo-Yusuf18 (2015)	South Africa	A secondary analysis of results from the randomized controlled trial (Louwagie et al.^17^)	409 randomized patients, current smokers, being ≥18 years, being on TB treatment for less than one month. Setting: primary care public facilities	To identify independent predictors of smoking cessation after adjusting for the intervention effect.	Smokers with high nicotine dependence were less likely to quit than those with low nicotine dependence during 1-month follow-up, but this effect was not sustained beyond this point in time. In multivariate analysis of the predictors of 7-day point prevalence abstinence during 1-month follow-up, motivational interviewing intervention was significantly effective only for those with nicotine dependence (OR=3.01; 95% CI: 1.74–5.21).	Potentially too small sample size to detect certain associations; self-reported smoking abstinence.
Sharma et al.^19^ (2018)	India	Open-label, randomized controlled study	Eight hundred adult patients enrolled (>18 years) with newly diagnosed sputum-positive pulmonary TB with self-reported history of current cigarette/rolled tendu or temburni leaf (bidi) smoking (more than 10 per day, every day for at least two months). Setting: DOTS centres	Primary outcome = a change in the TB score at 24 weeks and sputum culture conversion at week eight. Secondary outcomes = time to sputum smear conversion, weight gain at 24 weeks, number of patients who have quit smoking at 24 weeks, and mortality at 24 weeks.	Although intervention of nicotine replacement treatment (NRT) (nicotine gum 2 mg for smokers up to 25 bidis/cigarettes/day or 4 mg for those smoking more for 6 weeks) combined with counselling achieved higher quit rates (47.8% versus 32.4% for control group) and a greater reduction in TB score was seen in intervention arm, TB score did not meaningfully change at 8 and 24 weeks. Subsequent to treatment completion, most patients in both arms reported re-initiation of tobacco smoking (80.6% vs 79.7%).	The trial did not have a ‘drug treatment only’ arm without counselling. Also, the results of the study are ascribable to predominantly a single centre. The absent sputum production in a significant proportion of patients led to high loss to follow-up of the culture reports. Socioeconomic factors were not noted during the study.
Siddiqi et al.^20^ (2013)	Pakistan	Cluster randomized controlled study (the ASSIST trial)	n=1955 participants aged >18 years who were regular smokers of more than 1 cigarette or hookah session a day with suspected pulmonary TB. Setting: health centres registered as diagnostic centres by the TB program	Continuous CO-measured smoking abstinence at followup at 1 month and 6 months.	Behavioural support session (BBS) alone or with bupropion (BBS+) was effective in achieving continuous abstinence at 6 months for BSS+ (RR=8.2; 95% CI: 3.7–18.2), and for BBS alone (RR=7.4; 95% CI: 3.4–16.4).	Imbalances in the urban and rural proportions and smoking habits among treatment groups, inability to confirm adherence to bupropion treatment, and inability to validate longer-term abstinence or the effect of smoking cessation on TB outcomes.

**Table 2:** Summary and quality assessment of included non-randomized studies concerning smoking cessation/TDT in patients with TB of LMICs (N=28)

*Author, year*	*Country*	*Study design*	*Sample size/age/sex/type of sample*	*Study purpose*	*Findings*	*Limitations*	Selection*	Comparability*	Outcome*
Amara et al.^21^ (2008)	Morocco	Comprehensive cross-sectional telephone survey	75 respiratory physicians working in TB diagnosis and monitoring centres; 10.7% smokers (8/75), 58.7% men.	To evaluate attitudes and knowledge of Moroccan respiratory physicians towards smoking in the management of patients with TB and the feasibility of integrating smoking interventions into the national TB program. Of these, 84% still reported smoking status in patients’ medical records.	66.7% of those interviewed by phone were certain that smoking increases the incidence of TB, 96% believed that smoking would worsen the disease. More than 84% of the physicians inquired their patients about their smoking habits. Only 5.3% believed they were well trained to help smokers to stop.	Not reported	***		*
Aryanpur et al.^22^ (2016)	Iran	Crosssectional study	Newly-diagnosed PTB patients aged ≥18 years; self-reported smoking status; 248 (22%) patients were current smokers including 228 (20.2%) daily smokers and 20 (1.8%) occasional smokers. Setting: health centre	To determine the intention to quit and its associated factors among smokers newly diagnosed with PTB.	When diagnosed with TB, 59 smoker patients (23.8%) quit smoking. After PTB diagnosis, 99 patients (52.4%) had the intention to quit in the next month. Living in urban areas, office jobs, being single and a one unit increase in the motivation scale significantly increased the intention to quit smoking.	Cross-sectional nature of this study does not allow evaluation of the causal relationship between factors and smoking cessation, and its prognostic factors. Thus, further prospective studies are required. Meanwhile, as this study was conducted on patients with newly diagnosed PTB, its results cannot be generalized to all TB patients.	**		**
Awaisu et al.^23^ (2010)	Malaysia	Crosss-ectional study	n=817 newly diagnosed TB patients, 40.3% were smokers, 13.9% ex-smokers; 120 enrolled in the SCIDOTS project, 98.7% were males. Setting: TB clinics	To determine the prevalence of smoking among newly diagnosed TB patients and to evaluate the tobacco use knowledge and attitudes of those who are smokers in this population using a 58-item questionnaire.	Patients who were in the stage of contemplation/pre-contemplation had significantly less knowledge than those in the preparation stage of change (3.73 vs 5.38; p=0.004). 65.1% of patients believed that smoking is fun and 61.3% that it calms nerves. 70.1% of respondents also agreed or strongly agreed that smoking makes them relieve all life stresses. However, 87.5% of patients agreed or strongly agreed that: smoking is a waste of money, 91.3% that tobacco use is very dangerous to health; and 81.3% that smokers are more likely to die from heart disease compared with non-smokers.	The rates in study might have been grossly underestimated due to the unknown smoking status of a reasonable proportion of the newly diagnosed TB patients who might as well be tobacco smokers. A trend of underestimation when smoking prevalence is based on self-reports.	**		**
Awaisu et al.^24^ (2011)	Malaysia	Prospective non-randomized controlled intervention (the SCIDOT project)	n=80 current smokers at the time of TB diagnosis; 40 patients motivated to quit smoking (in preparation stage) received SCI in addition to DOTS (the intervention group and 46 unmotivated patients (in pre-contemplation and contemplation stages) received conventional DOTS regimen (the usual care group). SCI = eleven sessions of individualized cognitive behavioural therapy with (60%) or without (40%) nicotine replacement therapy (nicotine gum 2 mg and 4 mg, nicotine transdermal patch, and nicotine inhaler). Setting: respiratory clinics	To evaluate the impact of adding smoking cessation intervention (SCI) to conventional DOTS for TB on tobacco abstinence rates and TB treatment outcomes.	Subjects in the DOTS group were more dependent on nicotine than those in the SCIDOTS group (FTND, 5.43 ± 1.96 vs 4.32 ± 2.26; t = -2.439; p=0.017). At the end of 6-month follow-up, the one-month self-reported continuous abstinence rate, confirmed biochemically by both CO and saliva cotinine tests, was nearly 78% (31/40) in the intervention group versus 9% (4/46) in the usual care group (Pearson χ2=41.97; df=1; N=86; p<0.001).	The study compared persons motivated to quit smoking in an intervention group with those contemplating quitting smoking in a control group; therefore, the observed difference in smoking cessation between the groups would likely not have been so great.	***	*	**
Bam et al.^25^ (2015)	Indonesia	Cohort study	n=750 new smear-positive TB patients, 82.3% male, 77.6% current smokers. Of the 80 healthcare facilities, 52 (65 %) were tobacco-free in March 2011, the number of which increased to 80 (100%) by December 2012. Smoking was not permitted in any buildings, grounds or carparks. Cigarettes were not sold, and tobacco advertising, promotion and sponsorship were not permitted on the premises. Setting: health centres that provided DOTS services	To assess the implementation and effectiveness of the ABC smoking cessation approach for TB patients and the establishment of smoke-free environments in healthcare facilities and TB patients’ homes in Indonesia.	The point prevalence of the quit rate was 66.8% (389/582) at month 6. Predictors independently associated with quitting were the time from waking to the first cigarette >30 min, having a smoke-free home and the display of ‘no smoking’ signage at home at month 6. The ABC smoking cessation intervention was effective for: i) creating 100% tobacco-free health services, ii) promoting quitting smoking (66.8%), and iii) establishing smoke-free environments at home (86.1%).	Self-reporting of smoking status although the status of the patients’ smoking and of their smoke-free home were validated with a family member at month 6. Issues of time constraints and high workload were raised at the initial training by the healthcare staff before the intervention; however, healthcare workers reported neither high workloads nor time constraints during the review meeting at months 2, 5, and 6.	***	*	***
Boeckmann et al.^26^ (2019)	Bangladesh and Pakistan	Qualitative study	n=6 healthcare workers in Pakistan and 2 in Bangladesh (5 men), ranging in age from 23 to 60 years; n=35 patients (34 males), age ranging from 18 to 60 years Setting: clinics	Findings of a multi-country qualitative process evaluation assessing barriers to and facilitators of implementation of smoking cessation behavior support in TB clinics in Bangladesh and Pakistan.	All patients report willingness to quit smoking and recent quit attempts. Individuals’ main motivations to quit include their health and the need to provide financially for their family. Behavioural regulation such as avoiding exposure to cigarettes and social influences from friends, family and colleagues are the main themes of the interviews. Most male patients do not feel shy admitting to smoking, for the sole female patient interviewee, stigma was an issue. Health workers report structural characteristics such as high workload and limited time per patient as primary barriers to offering behavioural support.	Sample size, gender bias	***		***
Brunet et al.^27^ (2011)	South Africa	Cross-sectional study	n=424 patients with suspected TB; mean age 39.5 (18–82) years, 67% male, 71% black African, 28% HIV infected, 36% reported having previously suffered from TB, 65% current smokers and 17% had previously quit smoking. Setting: clinics	1) To estimate the prevalence of tobacco smoking in patients with suspected TB; and 2) to measure the sensitivity and specificity of their self-reported smoking status using plasma cotinine as the reference standard.	The prevalence of current smoking was estimated at 54% (95% CI: 49–58%) by plasma cotinine and 57% (95% CI: 52–61%) by self-report. The sensitivity and specificity of self-reported smoking was 89% (95% CI: 84–93%) and 81% (95% CI: 75–86%), respectively, using plasma cotinine as a reference standard.	Potential mis-classification of exposure due to self-report. The use of cotinine concentration has its own limitations, including the detection of tobacco chewers and patients using NRT, and failure to detect those who had not smoked for >48 h.	***		***
Campbell et al.^28^ (2014)	Nepal	Prospective controlled intervention study	n=246 cigarette smokers with smear-positive pulmonary TB, aged >16 years. Setting: TB centre	Continuous abstinence for more than 6 months.	Brief simple (= advice given over approximately 10 min at the beginning of TB treatment by a staff member, repeated at 2 and 5 months into TB treatment) resulted in a significant number (39%) quitting the habit for 6 months (CO-validated abstinence) vs 0% in a control group without gender difference.	Allocation to intervention or controls was not randomized. A further weakness was the delay of just over 2 years between the original training and start of enrollment, which arose mainly as a result of administrative problems.	**	*	***
Deepak et al.^29^ (2012)	India	Community-based, cross-sectional study	n=202 former patients with TB who had completed TB treatment at least 6 months before the interview; mean age 48 years; 52% (106/202) of users of any form of tobacco, 33 smokers, 60 smokeless tobacco (SLT) users, 13 both forms. Setting: TB units	Semi-structured pre-tested interview schedule; abstinence, persistence and relapse rates at eight time-points in relation to diagnosis of TB and treatment completion were analyzed separately for SLT use and smoking.	The relapse rate of SLT use was much higher than that of smoking because most tobacco messages provided by doctors to patients were general in nature and focused on smoking. More tobacco and TB-specific cessation messages need to be given to these patients.	Self-reported tobacco use. The findings of the study are not generalizable to all patients with TB since only patients who accessed TB units and completed treatment were studied.	***		**
El Sony et al.^30^ (2007)	Sudan	Feasibility study	48 medical assistants (MA), all presented during the study; n=513 previously untreated male patients (new cases) who were enrolled in treatment for pulmonary TB were recruited (81% of current smokers in the intervention group vs 36% in the control group). Setting: primary and respiratory care centres	To examine the feasibility of adding a simple cessation intervention to standard health care services for TB patients. A secondary question assessed the outcomes of tobacco cessation intervention by measuring reported tobacco use rates among patients in the intervention centres at the beginning and end of 12 months of follow-up.	According to baseline questionnaire given to 48 MA prior to the trial, 10 (21%) of the 48 MA reported using some form of tobacco, with no significant difference between those in the control and intervention centres. Only 8% of participating MA reported that they did not allow smoking or snuff dipping (10%) at the health centre and 22 (46%) reported almost always advising their patients about tobacco. Of all tobacco users who were followed up, reported cessation rates increased at each intervention. A 53.6% (165/308) abstinence rate at the end of TB treatment among those who were enrolled in a tobacco cessation program vs 14.3% (6/42) in a control group.	No biochemical validation of the reported high cessation rates.	****	*	***
Gupte et al.^31^ (2018)	India	Mixed-methods study	n=377 patients (84% men) with TB who were current tobacco users (12% smokers only, 78% SLT users only, 10% dual tobacco users); 25 DOTS providers (80% women, mean age 38 years) from 27 NGOrun centres trained to provide brief advice and cessation support in line with Union guidelines49. Setting: NGO-run DOTS centres	To determine: 1) the number of centres that started implementing brief tobacco cessation programs; 2) the characteristics of TB patients who were current tobacco users, stratified by type of tobacco; and 3) tobacco use status, stratified by TB category, following tobacco cessation advice.	A progressive trend in quit rates was observed during the treatment period (32% among new patients and 15% among those on retreatment, although the quality of documentation related to the brief advice and cessation support provided by DOTS providers declined. DOTS providers also felt that they had acquired the necessary skills needed to implement the intervention and suggested important recommendations including refresher training courses, the possibility of referring difficult patients and/or people with multiple addictions to experts and the need to simplify documentation.	Self-reported tobacco use status, study design.	***		***
Kanakia et al.^32^ (2016)	India	Crosssectional study	n=424 presumptive patients with TB aged >18 years, mean age 44 years (SD=16), smokers or smokeless tobacco users. Setting: tertiary care hospital	To assess the burden of tobacco use among presumptive TB patients and their willingness to avail of tobacco cessation services at a tertiary care hospital.	41.5% (176/424) use tobacco in previous 1 month (95% CI: 36.9–46.3%), 75% were smokers, 25% SLT form; 53% were willing to avail themselves of tobacco cessation services, if provided; the willingness was higher among those who had attempted to quit and failed in the past 1 year.	Not reported	***		**
Kaur et al.^33^ (2013)	India	Intervention study	n=2879 TB patients (81.7% with pulmonary TB), 1986 males (69%), registered for DOTS treatment; 46.3% (1333/2879) of TB patients were current users of tobacco – smokers and/or smokeless tobacco users, 89.6% males, 40.8% tobacco users resided in urban areas, and 52.2% were from rural areas. Setting: primary healthcare services	The possibility and outcome of integrating incorporating ‘brief advice’ in tobacco cessation intervention in TB patients who are registered for treatment under a TB control programme and are tobacco users.	While 35.9% of the TB patients were smokers, 39.1% used smokeless tobacco. 61.9% of males and 54.3% of females expressed their willingness to quit. At the end of 6 months, 67.3% of patients who were offered brief advice by the DOTS provider and the same advice was repeated during each interaction with the TB patient during the treatment period, quit tobacco, while 18.2% re-lapsed and 14.5% were lost to follow-up.	The confirmation regarding quitting of tobacco use was subjective assessment based on the self-statement by the patients. It was not validated by performing tests such as urine cotinine or carbon monoxide analysis of breath.	***		**
Lam et al.^34^ (2013)	South Africa	Cross-sectional study	n=707 recently diagnosed TB patients, aged ≥18 years and diagnosed with TB within the 2 months prior to interview date; 46% men, 73% HIV-infected; 6% (46) current smokers, 38% (267) former smokers. Setting: hospital	Assessed the current and recent smoking prevalence in those with TB and TB-HIV co-infection among hospitalized adults with recently diagnosed TB.	138 former smokers were reclassified as current smokers upon reporting smoking within 2 months before TB diagnosis, resulting in 26% of current smokers (184). By categorizing smoking status solely based on participants’ self-reported status at the time of interview, the group of participants who were current smokers but quit at symptom onset were misclassified as former smokers, when in reality they were smoking at the onset of TB symptoms.	Not reported	****		***
Lin et al.^35^ (2015)	China	Prospective study	n=244 patients with TB, current smokers from rural China. Setting: public health TB clinics	To assess incorporation of smoking cessation intervention (providing information on the harmful effects of tobacco smoke and smoking and TB+ every follow-up visit with reinforcement health messages and advice to quit) into routine TB services.	A majority (81.6%) had made no attempt to quit before the diagnosis of TB, 95.9% of smokers were willing to quit. 66.7% (156/244) reported abstinence at month 6 = remain abstinent at the end of TB treatment.	Findings may not be applicable to female smokers, as there were none in the study. Smoking status was based on self-report. 20.1% of the TB patients assigned to the SCI were not seen at month 6. Lack of a control group.	***		**
Louwagie & Ayo-Yusuf^36^ (2013)	South Africa	Cross-sectional study	n=1926 [22% (420/1924) self-reported smokers (37.6%) males] over 18 years of age seeking TB treatment. Setting: TB clinics	Semi-structured questionnaires	About half (51.8%) of current smokers had previously attempted to quit in the past 12 months (median quit duration 21 days), but very few patients had made use of cessation aids or services. The majority of respondents believed tobacco smoking was harmful for their health (90.5% were aware of the risk of lung cancer), but the level of awareness regarding the risk of stroke and heart attack was lower (48.5% and 38.2%, respectively), 40% noted that smokers were more likely to get TB and a third observed that smoking worsens TB, and were highly motivated to quit (median score 9).	The population of TB patients is not representative of all TB patients in the province or South Africa.	***		**
Louwagie et al.^37^ (2019)	South Africa	Mixed-method study	n=45 patients with TB (82% men, mean age 39.8 years), who smoked, drank alcohol or did both, and had not been treated for TB for more than one month. Setting: clinics	Semi-structured questionnaire to test the feasibility of the ProLife programme (a brief motivational intervention and SMS-programme) by monitoring fidelity to MI sessions and assessing the proficiency of LHW in facilitating the MI sessions.	Multiple risk behavior interventions similar to the ProLife programme can be effective and sequentially addressing smoking alongside other interventions as used in ProLife is preferable to simultaneous interventions. Most patients rated the MI sessions as helpful, ascribed positive attributes to their counselors, and reported behavioral changes. LHW: a) grasped the basic MI spirit but failed to understand specific MI techniques due to insufficient specific practice, b) viewed SMSs favourable, and c) considered limited space and privacy at the clinics as key challenges.	Small sample size. Social desirability bias may have led to overly positive feedback from both LHW and TB patients: Self-reported reductions in drinking and smoking were not validated.	**		
Mariappan et al.^38^ (2016)	India	A community-based cross-sectional study	n=235 patients with pulmonary TB; 83 (35.3%) smokers at the time of diagnosis, 23 (9.8%) used SLT. Setting: primary health care centres	To assess the prevalence and pattern of tobacco use among pulmonary TB patients residing in urban Puducherry and to study the association of various sociodemographic factors with current tobacco smoking and current smokeless tobacco use.	30 patients quit smoking and Self-reported on tobacco use. 22 patients reduced their smoking status after being diagnosed with TB and the rest 37.3% (31/83) maintained their smoking status. Male patients and having lower education were significantly associated with current smoking during TB treatment.	Self-reported on tobacco use.	**		**
Navya et al.^39^ (2019)	India	Mixed-methods study including a quantitative (cohort study)	n=413 patients; 278 (67.3%) males, mean age 42.6 years; 335 (81.1%) with pulmonary TB, 320 (77.5%) new TB cases, smokers and/ or smokeless tobacco users. Setting: TB units treated under Revised National Tuberculosis Control Programme	To: 1) report the extent of documentation of tobacco and alcohol usage data in the TB treatment card, and 2) explore the process, facilitators and challenges in the linkage of services for tobacco cessation and alcohol abuse from the perspective of health care providers and adult patients with TB.	The documentation of the tobacco use status was good but not universally done. Tobacco use was documented in 322 78%) of the TB treatment cards reviewed. Among the 86 (21%) patients documented as current tobacco users, 16 (19%) were linked to tobacco cessation services while no linkage was documented in the treatment cards of 46 (53.3%) patients.	All of the new TB treatment cards were not available for data collection in the few Tuberculosis Units of Dakshina Kannada district. This would have led to an over- or under-estimation of the results. Data could be validated for only a small subset of the total study population due to challenges in the two steps for obtaining informed consent.	**		**
Ng et al.^40^ (2008)	Indonesia	Cross-sectional study	n=239 male TB patients who completed the DOTS-based treatment regimen; 218 (91.2%) were ever smokers who had ever smoked even a puff of a cigarette. Setting: lung clinics	Study: 1) documents smoking patterns among TB patients before diagnosis, during treatment and post treatment; 2) identifies messages that health professionals and DOTS providers give their patients about smoking; and 3) identifies factors associated with smoking relapse among TB patients.	Only 11% (n=8) remained daily smokers while on TB treatment. Relapsed smokers were significantly younger and started to smoke their first cigarette earlier than quitters, more often than quitters perceived that smoking causes TB (p=0.01). Only one-third of ex-TB patients reported that they had been advised to quit smoking by a nurse, 69.2% received such advice from a doctor. Receiving a cessation message from one’s doctor was non-significantly associated with a lower likelihood of smoking relapse (OR=0.60).	Potential of recall bias when asking ex-TB patients about smoking levels. Our intention was to assess general levels of smoking at different points of time. Some of these former patients resumed smoking at the same levels as they did prior to illness, while others smoked at lower levels, mistakenly thinking that smoking at such levels is relatively safe.	**		***
Nichter et al.^41^ (2016)	Indonesia	Prospective two-arm intervention study	n=87 newly diagnosed male TB patients who smoked undergoing DOTS for TB at home. Setting: home	To assess the impact of TB-specific quit smoking messages in the TB clinic and at home.	Although most patients with TB quit smoking when undertaking treatment, nearly a third resume smoking when treatment is completed and this percentage increases to 40% six months later. Many former patients and their family members do not consider low-to-moderate level smokers to be real smokers, particularly those who have reduced their smoking from one to two packs a day to just a few sticks.	Self-report; small sample size.	***	*	***
Pradeepkumar et al.^42^ (2008)	India	Cross-sectional study	n=215 patients with TB (males); mean age 49.0 years; 94.4% (203/215) were ever tobacco users (smokers and/ or smokeless tobacco users). Setting: TB units	1) To document tobacco use patterns among TB patients at different time points before diagnosis, during treatment and following treatment; 2) to examine how often cessation messages are given to TB patients by health staff and DOTS providers; 3) to investigate how the messages received are understood; and 4) to identify critical points of time when cessation messages need to be given based on when relapse is most likely to occur.	79% quit within 1 week of diagnosis. During treatment period there were only 12.4% of persistent smokers and 87.6% of initial quitters of whom 64.2% stayed permanent quitters at the end of 6 months. Of the 48 relapsed TB patients, one third relapsed within the first 2–3 months of treatment, another third within the next 3 months of treatment and 21% within the 3 months of treatment. More than half of all relapses occurred during the early months of treatment among patients whose DOTS providers were non-health staff. Among patients who had DOTS providers who were health staff, most relapses occurred after completion of treatment.	The data are robust enough to support the conclusions about the need for: 1) repeated exposure of TB patients to smoking cessation intervention, and 2) interventions for former and quitting smokers to encourage sustained cessation.	**		***
Sereno et al.^43^ (2012)	Brazil	Mixed-methods study	n=16 DOTS providers and supervising physicians; 15/16 women, mean age 45.8 years; 20 patients, current smokers, completed the follow-up questionnaire and urine testing for cotinine. Setting: primary health care centres	A pilot study to determine whether DOTS workers could be trained to deliver smoking cessation counselling and referral interventions, to identify potential barriers to a full-scale randomized controlled trial on the effectiveness of integrated smoking cessation in DOTS, and to determine whether TB patients who smoke would agree to participate in such a program.	Their self-rated ability to communicate the 5 As improved significantly between pre-training and post-training, and to provide smoking cessation support improved without statistical significance. There is a dose-response relation between the session length of person-to-person contact and successful treatment outcomes. However, even minimal interventions lasting less than 3 min increase overall tobacco abstinence rates. Person-to-person treatment delivered for four or more sessions appears to be especially effective in increasing abstinence rates.	The confirmation regarding quitting of tobacco use was subjective assessment based on the self-statement by the patients. It was not validated by performing tests such as urine cotinine or carbon monoxide analysis of breath.	**		*
Shangase et al.^44^ (2017)	South Africa	Qualitative research design	20 inpatients (15 men) at a TB hospital who self-identified as smokers and had drug-resistant (DR) TB. Age ranged from 18 to 70 years. Setting: hospital	What are the barriers to smoking cessation among DR-TB inpatients in South Africa?	Using smoking as a coping mechanism was identified as an addiction-related barrier (for more details, see Table 3). Lack of access to smoking cessation interventions is a key structurallevel barrier highlighted in this study.	This study recruited participants who were inpatients and were being treated for drug-resistant tuberculosis; therefore, the generalizability of the results to outpatients is limited.	***		***
Shin et al.^45^ (2012)	China	Qualitative study	Randomly selected hospitalized patients from an inpatient registration list and conveniently selected patients attending an outpatient clinic for screening, aged ≥18 years, had begun treatment for TB in the past 6 months, and had smoked any time during the 30 days prior to diagnosis with TB. Two focus group discussions of 17 TB physicians and five focus groups of 39 patients were conducted. Setting: hospital	To compare perceptions about smoking cessation among TB patients and their physicians.	Patients who were advised to quit smoking by their physicians after diagnosis with TB were likely to progress in their stage membership and attempt to quit smoking. Patients and physicians were concerned about the likelihood of smoking relapse after patients recovered from TB. Physicians had low levels of knowledge regarding the effect of smoking on TB. Many doctors, particularly those who smoked, did not view smoking cessation as an integral part of TB treatment.	The focus groups were conducted * in one TB hospital in Beijing, China. The findings may not be generalizable to TB patients and providers in other TB facilities. Many physicians, particularly those who were smokers, did not view smoking cessation as an integral part of TB treatment and did not believe that their patients would accept smoking cessation counselling.	*		**
Siddiquea et al.^46^ (2013)	Bangladesh	Cohort study	n=615 current smokers; 99% of men, mean age 38 years (range 16–77); final evaluation possible in 562 patients. Setting: peri-urban TB centres	To determine whether a modified version of The Union’s ABC guideline (5–10 min of brief advice to quit smoking) in Bangladesh was effective in promoting smoking cessation among TB patients and determinants associated with smoking cessation	Overall, 82% (464/562) of smokers had quit and the quit rate increased progressively from the first follow-up to the end of TB treatment (usually at month 6 or 8). Patients were considered to have quit smoking if they reported that they had not smoked tobacco in the past 15 days.	This was a pilot study and may have generated an exceptionally high level of enthusiasm for counselling that led to higher quit rates. Such enthusiasm may not be sustained over the long term if introduced more widely. Self-reporting of quitting.	***		***
Tsai et al.^47^ (2016)	Taiwan	Cross-sectional retrospective study	n=123 patients with TB at a rural district hospital (78% of men, mean age 61.4 years, 45 [46.9%] smokers before TB diagnosis). Setting: TB outpatient clinic in a local hospital	To evaluate and compare changes in cigarette smoking and health-promoting behaviours reported before and after TB diagnosis among adults in a disadvantaged region.	The percentage of participants who smoked decreased to 30.2% (29/123) after receiving or completing TB treatment, determinants for current health-promoting behaviours were chronic disease (b= –0-.25; p=0.005) and completion of TB treatment (b=0.23; p=0.007). A high prevalence of cigarette smoking and low levels of health-promoting behaviours were observed before the diagnosis and during or after completing TB treatment.	Limited generalisability (relatively uneducated patients from rural hospital were recruited), design of the study and self-reporting of certain health-related behaviours.	**		**
Warsi et al.^48^ (2019)	Banglades, Nepal, and Pakistan	Mixed-methods study	25 semi-structured interviews and 12 Focus Group Discussion across Bangladesh, Nepal, and Pakistan, and administered the adapted a UK National Centre for Smoking Cessation and Training questionnaire to 36 TB health workers (HWs) (100% in Pakistan and 95% in Bangladesh did not smoke).	A brief behaviour support intervention (a flip book, a leaflet and a poster) was tested with patients and health workers. Health workers received training in delivering a 15–20 min intervention.	Patients were not opposed to being approached about their smoking habit, although HW expressed concerns that women might not admit to tobacco use and that asking about tobacco use was a sensitive issue. HW ability to deliver tobacco cessation behavioural support to patients was hindered by a lack of knowledge about tobacco and TB interaction, low understanding of tobacco cessation, and poor patient communication skills. Additional barriers shown in Table 3.	Use of multiple data sources (health workers, policymakers and patients), multiple methods (focus groups, interviews and questionnaire) and drawing on the theoretical framework of COM-B to guide data collection and analysis.	***		***

* The Newcastle–Ottawa Scale (NOS) for Assessing the Quality of Nonrandomized Studies^11^.

**Table 3:** Facilitators of and barriers to smoking cessation/TDT in patients with TB of LMICs identified in included studies

*Facilitators*
Confirmed TB diagnosis more likely than other respiratory conditions^14^
Living in urban areas, office jobs, being single significantly increased the intention to quit smoking^22^
Combined interventions^13,19,20^
Brief, but repeated interventions, motivation, brochures^15-17,28,31^
Tobacco-free healthcare facilities^25^
Time from waking to first cigarette of >30 min, routine screening for smoking, having a smoke-free home and display of ‘no smoking’ sign at home, regular reminders and encouragement of by family members to quit smoking^25^
Willingness to quit – higher in those with previous attempt(s) in the past year^32^
Incorporating of training in brief advice into existing training of DOTS providers^33^
Personalized behavioural counselling incorporated into routine TB services^24,35,43,46^
High level of awareness regarding smoking risk for health^36^
Multiple risk behaviour interventions^37^
Repeated tobacco use intervention follow-up at a minimum of 6 months after end of TB treatment^30,43^
Providing intervention not only at a health facility but also on a daily basis at community level by health volunteers^46^
***Barriers***
High nicotine dependence^18^
Misclassification of current smokers as former smokers at the time of TB diagnosis (= quit smoking at the onset of TB symptoms^34^)
Limited space and privacy at the clinics^37^
Male gender, lower education^38^
Daily smoking of more than 15 cigarettes/bidis at the time of diagnosis^41^
Patients’ cards without the provision to include about brief advice on smoking cessation given which has been mentioned in the TB treatment guidelines; lack of coordination between the TB treatment programme and tobacco cessation^39,43^
Identified in patients with drug-resistant TB: Addiction-related personal barriers – initiating smoking as teenagers, craving for a cigarette, smoking as part of the daily routine, failed quit attempts (relapses when feeling better) Structural (institutional) factors – lack of impact of health education sessions, lack of extramural activities when on hospital admission, lack of access to smoking cessation interventions (unaware of any available aids to stop smoking or NRT), easy access to cigarettes within a hospital setting (from staff, peers, visitors, shops close to hospital, hospital café)^44^ Non-addiction-related personal barriers – lack of knowledge about quit strategies, lack of willpower to quit, psychosocial stress, peer smokers’ influence
Barriers for HW to provide BSS: institutional lack of resources (insufficient space, high patient load, no reporting/recording of tobacco, overwork) and an absence of professional support through monitoring and evaluation^48^
***Possible barriers to smoking cessation/TDT – patients’/staff’s knowledge, attitudes***
Lack of resources (human, financial), low level of education of health providers on smoking cessation^21^
Beliefs that smoking is fun, calms nerves, relieves all life stresses^23^
Stigma (especially in women to admit using tobacco)^26,48^
Tolerance of smoking or snuff dipping at a health centre by medical assistants providing SCI, smoking staff^30^
Not considering low-to-moderate level smokers to be real smokers, particularly those who have reduced their smoking from one to two packs a day to just a few sticks^41^
Less knowledge that smoking increases risk of stroke and heart attack^36^
Physicians’ low levels of knowledge regarding the effect of smoking on TB – particularly physicians who smoked did not view smoking cessation as an integral part of TB treatment^45^
Decrease of initial enthusiasm for counselling (seen in pilot studies) over the long-term if introduced more widely^46^
Relying on the fact that the diagnosis of TB alone will lead to a more significant decrease in the prevalence of smoking among patient with TB^47^

**Table 4:** Factors associated with smoking relapse among TB patients in LMICs (N=6) identified in included studies

Short duration of pharmacotherapy^19^
Socio-cultural influences (i.e. family/friends smokers)^26^
Brief advice focused only on smoking could lead to a higher rate of SLT relapse seen as a form of harm reduction^29^
Receiving a disease-specific cessation message – associated with a lower likelihood of smoking relapse^40^
Perception of low-moderate level smoking as harmless^41^
Period of follow-up: increase in relapses within the 6 months of treatment and within the 3–6 months following treatment^40-42^

### Study quality

The inclusion criteria were met by 8 RCS, two of which were secondary analyses of data obtained from the selected studies (Table 1). Low risk of bias was assessed in 7 of 8 RCS (Table 5). The inclusion criteria were met by 28 non-randomized studies of variable methodological quality (Table 2).

**Table 5:** Risk of bias assessments assessed in randomized controlled studies

*Author, year*	*Random sequence generation (selection bias)*	*Allocation concealment (selection bias)*	*Blinding participants/personnel (performance bias)*	*Blinding outcome assessment (detection bias)*	*Incomplete outcome data (attrition bias)*	*Selective reporting (reporting bias)*	*Other bias*
Aryanpur et al.^13^ (2016)	Low	Low Low	Low	Low	Low	Low
Goel et al.^15^ (2017)	Low	Low Low	Low	Low	Low	Low
Kumar et al.^16^ (2017)	Low	Low Low	High	Low	Low	High
Louwagie et al.^17^ (2014)	Low	Low Low	Low	Low	Low	Low
Sharma et al.^19^ (2018)	Low	Low Low	Low	Low	Low	Low
Siddiqi et al.^20^ (2013)	Low	Low Low	Low	Low	Low	Low

### Facilitators of smoking cessation/TDT in patients with TB of LMICs

Facilitators of smoking cessation/TDT in patients with TB of LMICs are summarized in Table 3.

The most frequently described facilitators that foster smoking cessation/TDT in TB patients were repeated brief interventions (including motivation and brochures)^15-17,28,31^ followed by personalized behavioural counselling incorporated into routine TB services^24,35,43,46^. The SCIDOT project showed that adding smoking cessation intervention (SCI) to conventional DOTS increased biochemically validated 6-month abstinence^24^. RCS assessing behavioural intervention considered it an effective part of treatment increasing the success rate in quitting:

- The study of Louwagie et al.^17^ from South Africa reported higher efficacy of a brief motivational interviewing session (15–20 min) provided by lay healthcare workers (LHW) compared to only receiving standardized smoking cessation messages from a TB nurse and a smoking cessation booklet (control group).
- Smokers who received smoking cessation intervention (SCI) from a TB health visitor trained to deliver the ABC package (A=ask, B=brief advice, C=cessation support) were more likely to quit smoking compared to those who received existing standard of care in TB case management in India^15^.
- In the study by Kumar et al.^16^, the quit rate in the intervention group (physicians’ advice) was higher compared to the control group, but with no significant difference. However, the study evaluated only a 1-month abstinence period in a relatively small sample.

Three RCS studies confirmed the efficacy of combined interventions (behavioural support plus pharmacotherapy) in achieving longer-term abstinence at six months:

- The ASSIST study compared the success rates in a group treated by behavioural support (30-min consultation to encourage patients to plan smoking cessation 1 week later, and a 10-min session to review progress) combined with 7 weeks of bupropion therapy (75 mg/day during week 1, and 150 mg/day thereafter) to behavioural support only and usual care^20^.
- A similarly combined intervention that included behavioural therapy plus medical treatment with bupropion 150 mg/day during week 1, increased to 300 mg/day through week 9 led to success rates of 71.7% vs 33.9% for the brief advice group vs 9.8% for a control group receiving only the short-course, directly-observed treatment (DOTS) regimen (p<0.001)^13^.
- Biochemically verified quit rates were higher in the intervention arm (6 weeks of nicotine gum after which both arms received the same counselling) than in the control arm (47.8% vs 32.4%, p<0.001)^19^.

Two studies showed efficacy of repeated tobacco use intervention follow-up at a minimum of 6 months after end of TB treatment^30,43^.

In a secondary analysis of the ASSIST study^20^, Elsey et al.^14^ concluded that patients diagnosed with TB were more likely to be abstinent than those diagnosed with other respiratory conditions.

Some smokers’ characteristics were found to be facilitators too: willingness to quit – higher in those with previous attempt(s) in the past year^32^, time from waking to the first cigarette of >30 min, having a smoke-free home, display of ‘no smoking’ sign at home^25^, and a high level of awareness regarding smoking risk for health^36^. Other identified facilitators were: living in urban areas, office jobs, and being single, which significantly increased the intention to quit smoking^22^.

Facilitators originating in the healthcare system were the following: incorporating training in brief advice into existing training of DOTS providers^33^, multiple risk behavior interventions^37^, and tobaccofree healthcare facilities^25^. Finally, providing intervention not only at a health facility but also on a daily basis at community level by health volunteers facilitated smoking cessation^46^.

### Barriers to smoking cessation/TDT in patients with TB of LMICs

Barriers to smoking cessation/TDT in patients with TB of LMICs are presented in Table 3.

Male gender, lower education, high nicotine dependence and daily smoking of more than 15 cigarettes/bidis at the time of diagnosis, may be marked as non-influenceable risk factors that are negatively associated with success in smoking cessation/TDT^18,38,42^. Possible patients’ barriers to quit smoking may be less knowledge that smoking increases risk of cardiovascular diseases^36^ and their beliefs that smoking is fun and helps to deal with stress^23^.

The qualitative study of Shangase et al.^44^ conducted in patients with drug-resistant TB in South Africa found 2 categories of barriers to smoking cessation in patients with drug-resistant TB: 1) personal factors including addiction-related barriers (initiating smoking as teenagers, craving for a cigarette, smoking as part of the daily routine, failed quit attempts – relapses when feeling better) and non-addiction-related barriers (lack of knowledge about quit strategies, lack of willpower to quit, psychosocial stress, peer smokers’ influence); and 2) structural (institutional) factors (lack of impact of health education sessions, lack of extramural activities when on hospital admission, lack of access to smoking cessation interventions (unaware of any available aids to stop smoking or NRT), easy access to cigarettes within a hospital setting (from staff, peers, visitors, shops close to hospital, hospital café).

Several barriers were connected to the healthcare system itself: limited space and privacy at the clinics^37^, lack of coordination between the TB treatment programme and tobacco cessation^39,43^, lack of resources (human, financial), low level of education of health providers on smoking cessation^21^, tolerance of smoking or snuff dipping at a health centre by medical assistants providing SCI, and smoking staff^30^.

Regarding HW, barriers to provide BSS were as follows: institutional lack of resources (insufficient space, high patient load, no reporting/recording of tobacco, overwork), an absence of professional support through monitoring and evaluation^48^ or misclassification of current smokers as former smokers at the time of TB diagnosis (= quit smoking at the onset of TB symptoms)^34^. Low level of education of health providers on smoking cessation^21^, e.g. relying on the fact that the diagnosis of TB alone will lead to a more significant decrease in the prevalence of smoking among patients with TB^47^, and decrease of initial enthusiasm for counselling over the longterm^46^, may be barriers to provide SCI.

Staff knowledge regarding the effect of smoking on TB, as well as attitudes to smoking cessation may have an impact in providing smoking cessation/TDT within TB care^45,48^. Shin et al.^45^ in their qualitative study pointed out that mainly those physicians who smoked did not view smoking cessation as an integral part of TB treatment. Other barriers were not considering low-to-moderate level smokers to be real smokers, particularly those who have reduced their smoking from one to two packs a day to just a few sticks^41^, and less knowledge that smoking increases risk of stroke and heart attack^36^.

Another important barrier is mentioned in the Boeckmann et al.^26^ and Warsi et al.^48^ studies – asking about tobacco use was perceived a sensitive issue, especially among women who may not admit to tobacco use.

Table 4 shows factors associated with smoking relapse among TB patients in LMICs. Ng et al.^40^ conducted qualitative interviews with TB patients and concluded that the majority of daily smokers quit smoking when diagnosed with TB; however, over one in three of those patients relapsed within 6 months of TB treatment completion. Similarly, Pradeepkumar et al.^42^ and Nichter et al.^41^ found that most relapses occurred after completion of treatment. Short duration of pharmacotherapy might influence the number of patients who quit smoking, according to the randomized controlled study of Sharma et al.^18^. Possessing a disease-specific cessation message and focusing on all forms of tobacco use may decrease the probability of tobacco-dependence relapse^29,40^.

## DISCUSSION

### Statement of principal findings

The studies included in this narrative literature review highlighted the feasibility and efficacy of incorporating brief advice as well as specialized treatment for smokers into TB care. Based on the findings from the selected studies and identification of factors affecting successful quitting, we propose a summary of implications for practice regarding smoking cessation or TDT in patients with lung TB in LMICs in the following paragraphs.

Tobacco-free TB centres and non-smoking staff are a condition sine qua non to support their patients in quitting. The centres’ staff members should have an opportunity to treat their potential tobacco dependence or not to smoke during their working hours (e.g. to reduce withdrawal symptoms by nicotine replacement therapy)^25^.

The staff should understand the importance of quitting smoking for TB prognosis, of providing brief interventions, of their own non-smoking status; regular refresher training session should be arranged and made available.

In TB centres, a brief, repeated and empathic intervention should be provided to all patients with TB who smoke. It is possible that a majority can quit smoking when diagnosed with TB^40^ or at the time of onset of TB symptoms^34^. Support for these patients should be part of routine care. They would benefit from regular monitoring of their withdrawal symptoms (WSs) and from an offer of pharmacotherapy in the case of intensive WSs, and brief support at each session within their respective TB centres. Motivational intervention should increase the number of patients quitting in the future. Empathic intervention increases the probability that the majority of patients will admit smoking. A potential gender barrier should also be taken into account^26^.

While it may seem unnecessary to measure CO levels in patients with suspected TB at the first consultation to verify their smoking status^27^, such a strategy could help as bio-feedback for patients who are quitting.

All patients in TB centres who smoke should be educated, through a brief intervention, about the risks of smoking associated with their disease and the importance of the ‘not-even-one-puff’ rule, because a higher level of awareness implies a higher probability of quitting and a lower one a tendency to relapse^36,40^. Likewise, patients should be told that use of smokeless tobacco is a potential factor for smoking relapse^29^. Screening for smoke-free homes and recommendations to create them should be part of routine brief advice in smoking cessation.

Health volunteers may play a most important role in daily provision of intervention outside of health facilities directly in patients’ communities^46^.

Tobacco-dependence treatment ranges from brief advice to intensive behavioural support together with pharmacological treatment^7^. It has been proven by RTCs that behavioural support combined with first-line pharmacotherapy (bupropion and nicotine replacement therapy) is effective in achieving prolonged abstinence at six months in adult TB patients participating in TB programmes in LMICs^13,19,20^. One of the possible less expensive pharmacotherapy options is cytisine, whose efficacy compares to that of currently licensed products. A study from Bangladesh^8^ may bring interesting findings; two face-to-face behavioural support sessions will be delivered and cytisine (active arm) or matching placebo (control arm) will be administered in a 25-day course. The success rate may depend on pharmacotherapy duration (at least 3 months) as well as on the type of intervention provided. A brief motivational session (15–20 min) and the ABC approach (A=ask, B=brief advice, C=cessation support) are more effective than only a smoking cessation message. The specialized TDT should be provided by a trained doctor, nurse, psychologist or another health care provider, with such a specialist ideally available in each TB centre^49^.

Several months’ follow-up after TB treatment completion with brief behavioural support and offer of pharmacotherapy in the case of a lapse or early relapse should be available as prevention of relapse in abstaining patients. A high number of patients relapsed 3–6 months after completion of TB treatment^40,42^.

It is critical that national policymakers support effective tobacco control policies developed by the World Health Organization (WHO) Framework Convention of Tobacco Control (FCTC). According to the WHO MPOWER policy package, the main effective strategies designed to reduce the prevalence of tobacco use in the general population include those affecting the availability of tobacco products (taxes, advertising), minimizing exposure to tobacco smoke (smoke-free public indoor spaces without exception) and offering specialized help. Limiting bans on tobacco advertising and the ease of access to cheap tobacco, often in a single cigarette form, enhance barriers to quit^50^.

### Limitations and strengths

Although we conducted quality assessment of eligible studies, this is not a systematic review. Heterogeneous study designs made it impossible to conduct a metaanalysis and to assess the importance of individual factors in the quit process. However, this review maps and summarizes the available data on the topic, so that the formulated barriers and suggested facilitators can be identified for clinicians and policymakers to use. This summarized evidence could facilitate implementation of TDT in patients with TB in LMICs as an underused opportunity to significantly improve TB treatment outcomes and overall health.

### Implications for policy, practice and research

Effective TDT provided by trained healthcare professionals is based on the same pillars, regardless of country or socioeconomic status. These include: a brief intervention as a routine part of healthcare; appropriately adapted information materials about the harmfulness of smoking in relation to TB and about the options in a smoking cessation programme; trained non-smoking staff in specialized centres; availability of pharmacotherapy; and regular follow-up. All these should be incorporated as a new standard into care of TB patients who smoke.

## CONCLUSIONS

Raising awareness of the health risks of smoking in patients with TB as well as health providers and routine offer of tobacco-dependence treatment in established system of TB treatment in tobacco-free healthcare facilities may significantly contribute to increasing the abstinence success rate in patients with TB. Due to its low current availability but high potential impact, it is crucial to consider TDT in patients with TB as a topic for future research.
